# Theory of Mind and the Whole Brain Functional Connectivity: Behavioral and Neural Evidences with the Amsterdam Resting State Questionnaire

**DOI:** 10.3389/fpsyg.2015.01855

**Published:** 2015-12-10

**Authors:** Antonella Marchetti, Francesca Baglio, Isa Costantini, Ottavia Dipasquale, Federica Savazzi, Raffaello Nemni, Francesca Sangiuliano Intra, Semira Tagliabue, Annalisa Valle, Davide Massaro, Ilaria Castelli

**Affiliations:** ^1^Research Unit on Theory of Mind, Department of Psychology, Università Cattolica del Sacro CuoreMilan, Italy; ^2^IRCCS, Don Gnocchi FoundationMilan, Italy; ^3^Department of Electronics, Information and Bioengineering, Politecnico di MilanoMilan, Italy; ^4^Università degli Studi di MilanoMilan, Italy; ^5^Department of Psychology, Università Cattolica del Sacro CuoreBrescia, Italy; ^6^Dipartimento di Scienze Umane e Sociali, Università degli Studi di BergamoBergamo, Italy

**Keywords:** resting state components, theory of mind (ToM), functional connectivity (FC), resting state fMRI (rfMRI), graph analysis

## Abstract

A topic of common interest to psychologists and philosophers is the spontaneous flow of thoughts when the individual is awake but not involved in cognitive demands. This argument, classically referred to as the “stream of consciousness” of James, is now known in the psychological literature as “Mind-Wandering.” Although of great interest, this construct has been scarcely investigated so far. Diaz et al. (2013) created the Amsterdam Resting State Questionnaire (ARSQ), composed of 27 items, distributed in seven factors: discontinuity of mind, theory of mind (ToM), self, planning, sleepiness, comfort, and somatic awareness. The present study aims at: testing psychometric properties of the ARSQ in a sample of 670 Italian subjects; exploring the neural correlates of a subsample of participants (*N* = 28) divided into two groups on the basis of the scores obtained in the ToM factor. Results show a satisfactory reliability of the original factional structure in the Italian sample. In the subjects with a high mean in the ToM factor compared to low mean subjects, functional MRI revealed: a network (48 nodes) with higher functional connectivity (FC) with a dominance of the left hemisphere; an increased within-lobe FC in frontal and insular lobes. In both neural and behavioral terms, our results support the idea that the mind, which does not rest even when explicitly asked to do so, has various and interesting mentalistic-like contents.

## Introduction

A classical topic of interest both for psychologists and philosophers has been the spontaneous and autonomous flow of thoughts that occurs when persons are awake, but not involved in cognitive demands. This topic has been termed Mind-Wandering (MW) in the psychological domain (Smallwood and Schooler, 2006; Mason et al., 2007; Gruberger et al., 2011).

The first attempt to conceptualize such a cognitive process can be traced back to William James and his famous concept of the “stream of consciousness” (William, 1892), that indicates the continuous course of thoughts and feelings without a logical structure. A typical example is the interior monolog, used in psychological novels, such as the famous “Molly’s monolog” in the novel “Ulysses” by James Joyce (1922). Over the past century, the construct of MW has been identified by different terms: “day-dreaming” (Giambra, 1979), “task-unrelated images and thoughts” (Giambra and Grodsky, 1989), “stimulus independent thought” (Teasdale et al., 1995), “task-unrelated thought” (Smallwood et al., 2003), “incidental self-processing” (Gilbert et al., 2005), “spontaneous thought” (Christoff et al., 2009) and “inner speech” (Morin, 2009). Despite the interest, as showed by the variety of terms created to identify it, the experimental study of MW has been largely neglected. As Gruberger et al. (2011) point out in their review, this may be due to the ontological features of the construct itself, i.e., the fact that MW occurs spontaneously and unintentionally, which makes it difficult to observe and measure it in the absence of external cues.

Initial observations of the activity of the resting brain occurred quite accidentally in the second half of the past century. Ingvar (1979, 1985) noted the presence of a specific and consistent pattern of neural activation during the rest-task conditions, and that the most important areas involved in this network were the frontal regions. This idea of a specific neural network underlying the resting condition remained unexplored for several years, until the massive advent of neuroimaging techniques. The growing importance of cognitive neuroscience and the increasing use of its brain imaging methods, namely Positron emission tomography (PET) and functional magnetic resonance imaging (fMRI), have allowed the observation of the brain networks underlying MW. In fact, using PET and fMRI it was revealed that resting brain activity involves many areas (Hampson et al., 2002; Beckmann et al., 2005; Seeley et al., 2007; Greicius et al., 2009). In this context, the medial prefrontal cortex (mPFC), the posterior cingulate cortex (PCC) and the inferior parietal lobule (iPL) were consistently identified. Moreover, it was postulated that the hippocampal formation (HF) played a role as well, even if its involvement remained controversial for a long time because it depended on the specific aims of the studies (Buckner et al., 2008). A crucial contribution for the advances of the study of the MW and of its the neural basis came from Raichle et al. (2001), who coined the construct of the “Default Mode Network” (DMN), now a key construct in the study of the human brain activity. The scientific literature has identified the core regions involved in the DMN (Buckner et al., 2008): ventral mPFC, the posterior cingulate/retrosplenial cortex, the iPL, the lateral temporal cortex, the dorsal medial prefrontal cortex, and the HF. Most interestingly, the DMN comprises different components with specific functions that are active and interact in complex forms of cognition (for example creativity, Beaty et al., 2014), in particular in social cognition. Recent reviews and meta-analyses (see for example Schilbach et al., 2008, 2012; Mars et al., 2012; Li et al., 2014) have confirmed that the DMN is involved in the complex activity of social understanding, that comprises more specific dimensions such as self-consciousness, the self-other distinction, the self-other exchange (Schilbach et al., 2008), and the autobiographical processes (Andrews-Hanna et al., 2014). A crucial ability for a successful self-other distinction and self-other exchange is theory of mind (ToM), or mindreading or mentalizing, i.e., the ability to represent self and other’s mental states and to use such meta-representations to understand, explain, and foresee human behavior (Doherty, 2008). Over the past decades, the psychological and neuropsychological literature has consistently demonstrated that ToM typically develops during the preschool and school-age period, and that it undergoes continuous changes across the life-span. In particular, this ability, which is extremely important for social aspects of life, undergoes significant changes both on the behavioral and on the neural level in successful and unsuccessful neurocognitive aging (see for example Castelli et al., 2010, 2011; Baglio et al., 2012; Cabinio et al., 2015). As regards the most recent evidences concerning the neural basis of MW, a meta-analytic review by Fox et al. (2015) highlighted that focusing on the activation of the DMN alone may be overly reductive for an exhaustive description of the neural basis of spontaneous thought. In fact, they identified various regions, among which those that classically belong to the DMN (such as the medial prefrontal cortex/anterior cingulate cortex) and a series of regions outside the DMN (such as the secondary somatosensory cortex and the insula). Therefore, in the present study we decided to investigate the neural basis of MW following a whole-brain approach, i.e., looking also at areas beyond just the DMN.

Behavioral methods to study MW have been developed only recently (Gruberger et al., 2011) and they have been employed in association with brain imaging techniques. Three types of methods are deeply related to the brain imaging techniques: parametric modulation of self-relatedness, parametric modulation of cognitive load, and paradigm-free analysis of neuronal dynamics. In the first approach, neural activations during self-related tasks are compared to neural activations at rest, when MW is supposed to occur most frequently (see for example Gusnard et al., 2001). In the second one, the contrast between the task-condition and the resting-condition is the procedure of “the contrast of rest minus task.” The idea at the basis of such a method was to demonstrate that to a low cognitive load in a certain task condition corresponds a high activation in the DMN areas during the task (see for example Christoff et al., 2004; McKiernan et al., 2006; Mason et al., 2007). Finally, in the paradigm-free analysis of neuronal dynamics, no behavioral paradigm is devised, because the participants simply lie resting during the examination (see for example Horovitz et al., 2008, 2009).

Two types of methods, instead, place more attention on the behavioral evaluation of MW: real-time sampling, and retrospective evaluation. In the former, participants have to indicate whether or not they were experiencing a spontaneous thought (i.e., not-linked to task performance) when they heard an auditory stimulus during an fMRI scan session (see for examples McKiernan et al., 2006; Mason et al., 2007; Christoff et al., 2009). In the latter, a questionnaire is proposed after a resting session, without interrupting the MW experience, and asking the participants to recall the contents of the MW activity. Even if the use of self-report methods is largely employed in the psychological research, there is a paucity of studies regarding MW with such a method. The first and few attempts in the past years (Giambra, 1979; Klinger and Cox, 1987; Matthews et al., 1999) were not validated and thus did not find proper consideration in the neuroscientific literature. In recent years, three attempts to develop self-report measures of the resting activity deserve consideration. D’Argembeau et al. (2005) asked participants to rate the amount of thoughts experienced, regardless of their content, in a questionnaire immediately after the fMRI session. Delamillieure et al. (2010) devised the Resting State Questionnaire, which consists of 62 items that cluster five main types of mental activity: visual mental imagery, inner language, somatosensory awareness, inner musical experience, and mental manipulation of numbers. Participants retrospectively rate the proportion of time spent in each mental activity during the resting-state fMRI (rfMRI) acquisition using a 0–100% scale. Recently, Diaz et al. (2013) created the Amsterdam Resting State Questionnaire (ARSQ), a 27 items instrument, and administered it after a 5-min resting session in a non-fMRI session, in order to investigate the MW in a pure behavioral manner. This procedure has the advantage to limit the possible distortion due to the experimental peculiarities of fMRI acquisition. Through exploratory and confirmatory factor analyses (EFA and ECA), they identified seven factors: discontinuity of mind, theory of mind, self, planning, sleepiness, comfort, and somatic awareness. Stoffers et al. (2015) identified 11 positive associations between brain- functional network connectivity and ARSQ dimensions. Specifically, “Sleepiness”, “Visual Thought”, and “Discontinuity of Mind” appeared to be significantly associated with functional connectivity within the Visual, Sensorimotor and Default Mode networks.

The present study aims at evaluating for the first time the seven-factor structure of the ARSQ in an Italian sample, and at exploring the neural correlates of a subsample of participants divided in two groups on the basis of the scores obtained in the ToM factor. In particular, for the rfMRI analysis we used an approach well described in the literature (Achard et al., 2006; van den Heuvel et al., 2008; Bullmore and Sporns, 2009; Tyszka et al., 2014) to examine all the areas of the whole-brain network. Furthermore, since the literature has shown a partial overlap between areas associated with MW areas and those with the ToM network, particularly the mPFC/anterior cingulate cortex, we decided to focus on the possible correlation between the neural areas activated during the resting session and the respective ToM degree that has engaged the mind during the session itself.

## Materials and Methods

### Subjects

Data were obtained from 670 participants that completed the ARSQ. Most of our sample (*n* = 400) was obtained from students enrolled at the Catholic University of Milan and Piacenza. Moreover, students of other Universities in Italy were contacted using social network and mailing lists provided by student organizations (*n* = 193). Finally, we handed out the questionnaire to a sample of people (*n* = 70) already involved in another study at Don Gnocchi Foundation in Milan. According to the recommendations of the Declaration of Helsinki about ethical principles for medical research involving human subjects, both local ethics committee approval of the Don Gnocchi ONLUS Foundation and written informed consent from all subjects to participate in the study were obtained before study initiation.

### Amsterdam Resting State Questionnaire

On the basis of research by Diaz et al. (2013), we administered the ARSQ to test its structure and reliability in an Italian sample. Although an updated version of the questionnaire was recently published by the same research group (Diaz et al., 2014), it was not available when we started our study. The measurement of resting state through ARSQ is done in two steps: in the first, the participant is required to experience a resting state session, i.e., to remain alone for 5 min in a quiet and silent room, in a comfortable position with the eyes closed. Participants are also recommended to free the mind from all thoughts, and to set an alarm that will alert when the 5 min of the resting session have passed. In the second step, participants are required to fill out the questionnaire about sensations and thoughts experienced during the resting session.

The 27 items of the ARSQ and the five control items were translated into Italian with the back-translation procedure. The control items aim to evaluate whether the participants performed the questionnaire properly. Participants are asked to express their agreement with each item using a five point Likert scale: 1 = “Completely Disagree,” 2 = “Disagree,” 3 = “Neither Agree or Disagree,” 4 = “Agree,” and 5 = “Completely Agree.” Diaz et al. (2013) identified and confirmed a structure of seven factors: discontinuity of mind, theory of mind, self, planning, sleepiness, comfort, and somatic awareness (see **Table 1**).

**Table 1:** Control items and seven-factor structure of the ARSQ.

Control items	• I felt motivated to participate • I have difficulty remembering my thoughts • I have difficulty remembering my feelings • I had my eyes closed • I was able to rate the statements

Discontinuity of mind	• I felt restless • I had busy thoughts • I had my thoughts under control • I had rapidly switching thoughts • I had difficulty holding on to my thoughts

Theory of mind	• I thought about others • I thought about people I like • I place myself in other peoples’ shoes

Self	• I thought about my feelings • I thought about my behavior • I thought about myself

Planning	• I thought about my work/study • I thought about solving problems • I thought about the past • I thought about the future • I had deep thoughts • I thought about things I need to do

Sleepiness	• I felt tired • I felt sleepy • I had difficulty staying awake

Comfort	• I felt comfortable • I felt relaxed • I felt happy

Somatic awareness	• I thought about my health • I was conscious of my body • I thought about my heartbeat • I thought about my breathing

After dataset constructions we filtered the sample on the basis of the control items in order to ensure the inclusion of participants who had completed the resting state session before filling in the questionnaire. More specifically, the subjects who responded less than “agree” at least to one of the questions “I felt motivated to participate,” “I had my eyes closed” and “I was able to rate the statements” or less than disagree at least to one of the questions “I have difficulty remembering my thoughts” and “I have difficulty remembering my feelings” were excluded from the sample. We also removed those subjects who did not complete the entire questionnaire. The final sample consisted of 304 subjects (*M*_age_ = 29.26 years, *SD*_age_ = 11.04).

### MRI Acquisition Protocol

A sub-group of the whole dataset, which included 28 healthy right-handed subjects (mean age ±*SD* = 54.32 ± 18.79 years; range: 21–79 years; nine males), was acquired at Don Gnocchi Foundation, IRCCS Santa Maria Nascente (Milan, Italy), using a 1.5 T Siemens Magnetom Avanto (Erlangen, Germany) magnetic resonance imaging (MRI) scanner with an 8-channel head coil. All the subjects had no history of neurological, cardiovascular, or metabolic disorders and voluntarily participated in the study.

Resting state fMRI (rfMRI), BOLD EPI images were collected at rest for approximately 6.6 min (TR/TE = 2500/30 ms; resolution = 3.1 mm × 3.1 mm × 2.5 mm; matrix size = 64 × 64; number of axial slices = 39; number of volumes = 160). Subjects were instructed to keep their eyes closed, not to think about anything in particular, and not to fall asleep. High resolution T1-weighted 3D scans were also collected (TR/TE = 1900/3.37 ms; resolution = 1 mm × 1 mm × 1 mm; matrix size = 192 × 256; number of axial slices = 176) to be used as anatomical references for rfMRI analysis. After the rfMRI session, the ARSQ was administered, as in Diaz et al. (2013).

### rfMRI Data Analysis

Pre-processing of rfMRI data was carried out using FSL (Smith et al., 2004; Jenkinson et al., 2012). Standard pre-processing involved the following steps: motion correction with Motion Correction Linear Image Registration Tool (MCFLIRT – Jenkinson et al., 2002); non-brain tissues removal with Brain Extraction (BET; Smith, 2002); spatial smoothing with a 5 mm full-width at half-maximum (FWHM) Gaussian kernel; high-pass temporal filtering with a cut-off frequency of 0.01 Hz. Single-subject spatial independent component analysis (ICA) with automatic dimensionality estimation was performed using MELODIC (multivariate exploratory linear optimized decomposition into independent component) 3.0 (Beckmann and Smith, 2004). Subsequently, each subject’s dataset was cleaned from artifacts using the FMRIB’s ICA-based Xnoiseifier (FIX) toolbox (Griffanti et al., 2014; Salimi-Khorshidi et al., 2014), as it has been shown that FIX is a good approach for data denoising (Pruim et al., 2015) and is more effective than other approaches in detecting functional alterations in a small-sample population and using a clinical scanner (Griffanti et al., 2015). The 24 motion parameters (i.e., the six rigid-body time series, their backward-looking temporal derivatives and the squares of the twelve resulting regressors) estimated by MCFLIRT were also regressed out.

After the pre-processing, each single-subject 4D dataset was aligned to the subject’s high-resolution T1-weighted image using linear registration (FLIRT, Jenkinson and Smith, 2001; Jenkinson et al., 2002) with the brain-boundary registration cost function (BBR, Greve and Fischl, 2009), registered to MNI152 standard space using Non-linear Image Registration Tool (FNIRT, Andersson et al., 2007a,b), and subsequently resampled to 2 mm × 2 mm × 2 mm resolution.

#### Regions of Interest

Resting state fMRI signal was extracted from the regions of interest (ROIs) defined by anatomical parceling the cortical areas using the Harvard-Oxford atlas (Frazier et al., 2005; Desikan et al., 2006; Makris et al., 2006; Goldstein et al., 2007), as seen in previous studies (Davis et al., 2013; Tyszka et al., 2014). The maximum overlap discrete wavelet transform (Percival and Walden, 2000) was then used to decompose the time series into wavelet coefficients at four scales (scale 1: 0.0066–0.0075; scale 2: 0.0075–0.016 Hz; scale 3: 0.016–0.0364 Hz; scale 4: 0.0364–0.1 Hz) and estimate the wavelet-correlation matrices at each scale (Achard et al., 2006). We focused on scale 4 (0.0364–0.1 Hz), in which the spontaneous low-frequency oscillations of the BOLD signal are mostly located (Fransson, 2005).

The wavelet-correlation matrices were used for estimating possible functional connectivity (FC) dependencies with the ToM factor. Subjects were divided in two groups, i.e., high-ToM and low-ToM, each composed by 14 subjects, depending on the mean score (respectively higher or equal/lower than 3, which is the arithmetic mean across the possible ToM scores in the range: 1–5) that the subject obtained in the answers to the three items of the factor of theory of mind (“I thought about others,” “I thought about people I like,” and “I placed myself in other people’s shoes”).

Significant differences between the high-ToM and low-ToM groups were assessed with a two-sample unpaired *t*-test using the Network Based Statistic toolbox (NBS, Zalesky et al., 2010).

## Results

### Preliminary Confirmation of the Original 7-Factor ARSQ Structure

Descriptive analyses on the items were conducted. All the items showed a normal distribution (skewness and kurtosis < 1.5). In order to test the original structure that emerged from the ARSQ data analysis we performed a confirmatory factor analysis (CFA) with MPlus software 6.11 (Muthén and Muthén, 1998–2011) on the dataset with 27 items extracting in seven factors. The results confirmed that the original model adapts to the Italian data, showing acceptable indices of fit and parameters substantially in line with those of the original study (Diaz et al., 2013; see **Tables 2** and **3**). Composite reliability (Bagozzi, 1994) proved well-above 0.80 for all the factors except for “ToM” where the value was 0.66. All the items significantly saturated the respective latent factors. However, ten items did not saturate at more than 0.40, of which only one item of the factor “Somatic” showed a value lower than 0.2 (range: 0.17–0.88).

**Table 2:** Fit indices for the models in Diaz et al. (2013) and current study samples.

Models	X^2^ (*p*)	Df, *N*	RMSEA (90% C.I.)	SRMR	CFI
Diaz et al., 2013, #11685	2455.63 (<0.001)	303, 813	0.093	-	0.88
Present study	577.28 (<0.001)	303, 304	0.055 (0.048–0.061)	0.068	0.84

**Table 3:** Correlations among factors.

	DoM	ToM	Self	Plan	Sleep	Comfort	SomA
DoM	–						
ToM	0.58**(0.36)	–					
Self	0.51**(0.58)	0.13 (0.59)	–				
Plan	0.59**(0.58)	0.33**(0.70)	0.60**(0.21)	–			
Sleep	0.12 (0.32)	0.11 (0.11)	-0.04 (0.24)	0.22*(0.21)	–		
Comfort	-0.47**(-0.72)	-0.03 (0.04)	-0.07 (-0.27)	-0.24**(0.20)	-0.06 (-0.25)	–	
SomA	-0.15*(0.35)	-0.12 (0.27)	0.18*(-0.71)	-0.02 (0.48)	-0.00 (0.31)	0.09 (-0.30)	–

*^∗^*p* < 0.01, ^∗∗^*p* < 0.001.*

### Resting State fMRI

All the subjects completed the MRI session; no structural alterations were found. Age and sex matching between the two groups were respectively verified by means of two-sample unpaired *t*-test and Chi-squared test (*low-ToM*: mean age = 49.36 ± 18.22, three males, mean ToM factor = 1.86 ± 0.68; *high-ToM*: mean age = 59.28 ± 18.65 years, six males, mean ToM factor = 3.31 ± 0.36).

#### Wavelet-correlation Matrices

**FIGURE 1:**
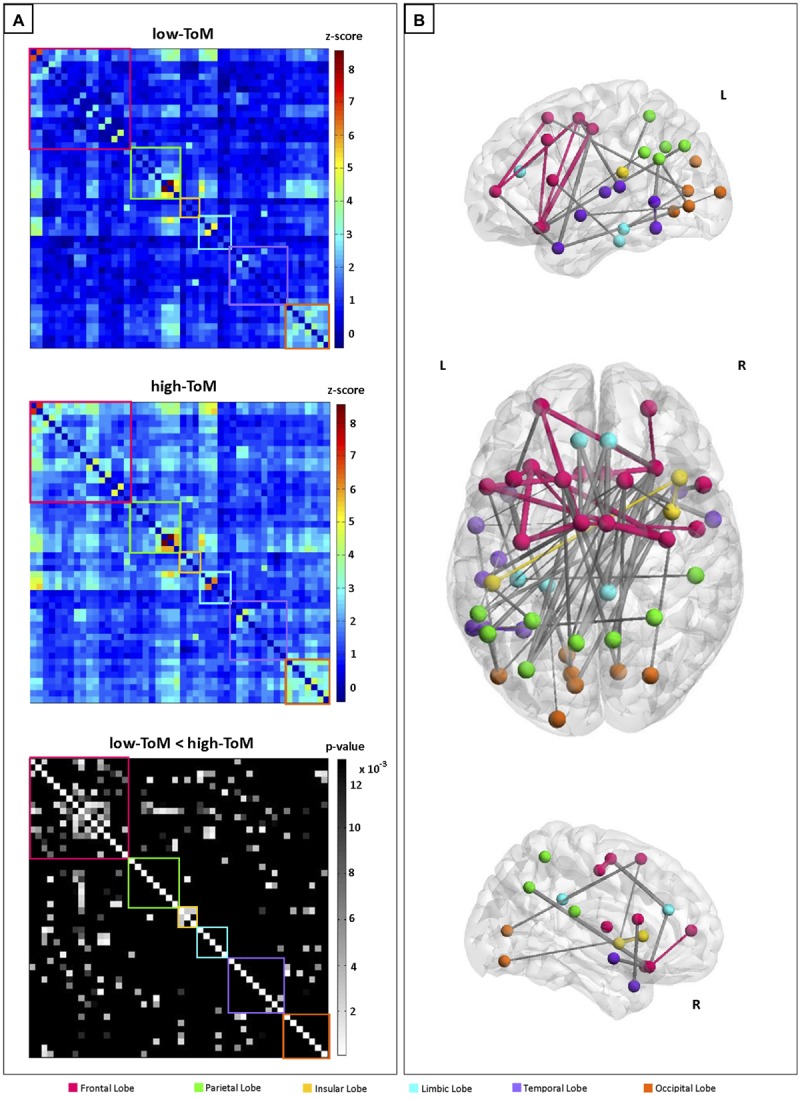
**(A)** Sub-network functional connectivity (FC) matrices among the 48 nodes (frequency range: 0.0364–0.1 Hz), averaged across the subjects belonging to the two groups: low-ToM (top) and high-ToM (middle); on the bottom, the statistical results for the contrast high-ToM > low-ToM (p_NBS-corr_ ≤ 0.013). Within-region FC is arrayed on diagonal blocks; between-region FC appears in off diagonal blocks. Colored boxes denote lobe membership: red = Frontal Lobe; green = Parietal Lobe; yellow = Insular Lobe; light-blue = Limbic Lobe; purple = Temporal Lobe; orange = Occipital Lobe. The color bars indicate the FC range (z-score: -1÷9) for the low-ToM and high-ToM connectivities matrices and the statistically significant p-values in grayscale (p_NBS-corr_ > 0.013 are black colored). **(B)** Graph representation of FC differences between the two groups. The edge size is proportional to the extent of FC difference between high-ToM and low-ToM. Within-region edges are represented with the color of their own region; between-region edges are colored in gray.

We evaluated the FC differences between the two groups with a two-sample unpaired *t*-test and identified a sub-network with higher values of FC in high-ToM compared to low-ToM. **Figure 1A** shows FC of the 48 sub-network nodes (see **Table 4** for details), expressed as the mean z-score across the subjects belonging to the low-ToM (top) and high-ToM (middle) groups. Six main regions were identified and highlighted with different colors along the diagonal: the frontal lobe (16 nodes, red box), the parietal lobe (eight nodes, green box), the insular lobe (three nodes, yellow box), the limbic lobe (five nodes, light-blue box), the temporal lobe (nine nodes, purple box), and the occipital lobe (seven nodes, orange box). The 48 nodes reported in detail in **Table 4**. To note, among those nodes it is possible to detect some core areas belonging to the DMN (medial frontal gyrus, iPLs, parahippocampal gyrus and precuneus) and to the salience network (insula and cingulate gyrus). The last row of **Figure 1A** reports the statistical results of the two-sample unpaired *t*-test (high-ToM > low-ToM, *p* < 0.05, NBS-corrected for multiple comparisons). **Figure 1B** shows a graph with the 48 nodes of the sub-network and the edges relative to a significant difference of FC between the two groups. This graph indicates a marked functional asymmetry between the left and the right hemispheres, as it shows more significantly different FC in the left hemisphere than in the right one. Globally, **Figure 1** highlights a greater FC variation in the frontal and insular lobes compared to the others.

**Table 4:** Characterization of the 48 cortical areas extracted from the Harvard-Oxford atlas with higher values of functional connectivity in high-ToM compared to low-ToM.

#	Coordinates (x, y, z)	Hemisphere	Lobe	Area
1	-25, 53, 8	L	Frontal	Middle frontal gyrus (BA10)
2	26, 52, 9	R	Frontal	Middle frontal gyrus (BA 10)
3	-15, 18, 57	L	Frontal	Superior frontal gyrus
4	15, 18, 58	R	Frontal	Superior frontal gyrus
5	-38, 18, 42	L	Frontal	Middle frontal gyrus
6	-51, 15, 15	L	Frontal	Inferior frontal gyrus (BA 44)
7	52, 15, 16	R	Frontal	Inferior frontal gyrus (BA 44)
8	-27, 24, -16	L	Frontal	Inferior frontal gyrus (BA 47)
9	29, 23, -16	R	Frontal	Inferior frontal gyrus (BA 47)
10	-34, -12, 49	L	Frontal	Middle frontal gyrus
11	35, -11, 50	R	Frontal	Middle frontal gyrus (BA 6)
12	-6, -3, 56	L	Frontal	Medial frontal gyrus
13	6, -3, 58	R	Frontal	Medial frontal gyrus (BA 6)
14	-6, 21, -16	L	Frontal	Subcallosal gyrus
15	6, 20, -16	R	Frontal	Subcallosal gyrus
16	49, -6, 11	R	Frontal	Precentral gyrus
17	49, -28, 22	R	Parietal	Inferior parietal lobule
18	-29, -49, 58	L	Parietal	Inferior parietal lobule
19	29, -48, 59	R	Parietal	Inferior parietal lobule
20	-55, -46, 34	L	Parietal	Inferior parietal lobule
21	-50, -56, 29	L	Parietal	Supramarginal gyrus
22	-8, -60, 37	L	Parietal	Precuneus
23	9, -58, 38	R	Parietal	Precuneus
24	-32, -73, 38	L	Parietal	Precuneus
25	41, 19, 5	R	Insular	Insula
26	38, 3, 0	R	Insular	Insula
27	-48, -32, 20	L	Insular	Insula (BA 13)
28	7, -36, 30	R	Limbic	Cingulate gyrus (BA 23)
29	-7, 37, 21	L	Limbic	Anterior cingulate (BA 32)
30	7, 36, 23	R	Limbic	Anterior cingulate (BA 32)
31	-22, -32, -17	L	Limbic	Parahippocampal gyrus (BA 36)
32	-36, -29, -25	L	Limbic	Parahippocampal gyrus (BA 36)
33	-45, -20, 7	L	Temporal	Superior temporal gyrus (BA 13)
34	-40, 11, -30	L	Temporal	Superior temporal gyrus
35	41, 13, -29	R	Temporal	Superior temporal gyrus
36	-56, -4, -8	L	Temporal	Middle temporal gyrus
37	57, -1, -10	R	Temporal	Middle temporal gyrus (BA 21)
38	-53, -30, 11	L	Temporal	Superior temporal gyrus (BA 41)
39	-57, -53, 1	L	Temporal	Middle temporal gyrus
40	-52, -53, -17	L	Temporal	Inferior temporal gyrus (BA 20)
41	-33, -54, -16	L	Temporal	-
42	-13, -66, -5	L	Occipital	Lingual gyrus (BA 18)
43	27, -75, -12	R	Occipital	Lingual gyrus
44	-9, -80, 28	L	Occipital	Cuneus (BA 19)
45	-17, -96, 7	L	Occipital	Cuneus
46	-45, -76, -2	L	Occipital	Middle occipital gyrus
47	-10, -75, 8	L	Occipital	Cuneus (BA 23)
48	12, -74, 8	R	Occipital	Cuneus (BA 23)

*#, Identification number; BA, Brodmann area; L, left; R, right.*

## Discussion and Conclusion

The present study aimed at confirming the factorial structure of the ARSQ and at exploring the neural correlates of the factor of theory of mind of the ARSQ thorough brain imaging techniques.

The results of the CFA have showed satisfactory fit indexes, supporting the seven factors structure originally found by Diaz et al. (2013). The confirmation of the seven factors structure contributes to measure the human mind activity, that appears to be restless even when persons are required to think about nothing. The results have also highlighted the presence of a statistically significant difference in the levels of the FC of 48 nodes between the subjects with a low mean in the factor of Theory of Mind (low-ToM) and the subjects with a high mean in the factor of theory of mind (high-ToM), i.e., subjects who report having thought a lot about other persons. In particular, our functional whole-brain network analysis showed that high-ToM subjects show more FC in the frontal lobe and in the insula, with a dominance of the left hemisphere.

The mPFC, a core region of the DMN, has been consistently identified as part of a circumscribed neural circuit underlying mentalizing (Amodio and Frith, 2006; Frith and Frith, 2006; Schurz et al., 2014). Moreover, our findings suggest that connectivity within the frontal lobes facilitates mentalizing ability. The evidence of frontal lobe involvement in ToM is in line with previous MRI results provided over the past years (see for example Stone et al., 1998), also by lesion studies: subjects with frontal lobes lesions show deficits in the solution of ToM tasks (see for example Rowe et al., 2001; Bird et al., 2004).

Without any intention to establish a causal relation between neural activation and psychological activity during resting state, it is possible to argue that the higher FC in the areas involved in the voluntary control of behavior, consciousness and executive function may depend on two reasons. In the first case higher FC may express the attempt to “control” the mental activity in order to comply with the experimental instruction “to free the mind from every kind of thoughts.” In the second case, the unrested mind involves itself in thinking about others by using the frontal lobe connectivity to decouple its own perspective from that of other’s.

Interestingly, our findings show that the FC changes are not limited to the areas belonging to the DMN, but are extended to include other networks. In fact, the insula is part of the “salience network,” which is impaired by damages to the frontal lobes. In terms of functionality, the insula mediates feelings linked with specific emotional states (Damasio et al., 2000; Critchley et al., 2004, 2005), revealing its role in binding cognition and emotion (Kurth et al., 2010). The insula is also involved in social emotion processing (Lamm and Singer, 2010), i.e., the affective states that we experience in the interaction with other people and that are related to the social context. It may be important to remember that the items of the factor of theory of mind were “I thought about others,” “I thought about people I like,” and “I placed myself in other people’s shoes” and that the insula has been found to be active in various type of ToM tasks, especially in the Eyes Test that evaluates the mindreading ability through eye gaze (Schurz et al., 2014). The insular cortex may also be considered the main cortical locus of an interoceptive system that regulates affective feeling states from the skin (Olausson et al., 2002; Loken et al., 2009). In the light of these evidences, the higher insular FC in High-ToM subjects may express the first-person experience with others in terms of affective and emotional feelings and in terms of proximal and proprioceptive involvement with them.

It is interesting to notice that the ability of mentalizing, as measured by the ToM factor in terms of “thinking about others,” implies on the neural level the involvement of brain areas linked both to the cognitive and the affective components, namely the frontal lobes and the insula, respectively. The functional asymmetry between the hemispheres with significantly higher FC in the left side can presumably be connected with the construct of narrative thought (Bruner, 1990). According to Bruner, ToM would be better understood if reworded in terms of autobiographical construction of the self. The latter is grounded on language that neuroscientific evidences posit as a prevalent left hemisphere activity.

This study has also some limitations. Although the CFA showed satisfactory parameters, the moderate reliability of the items of the ToM factor should suggest a cautious generalization of the results about the correlation between fMRI evidences and the ToM latent factor.

Future research will have to increase the number of items of the ToM factor. A greater number of behavioral details of the resting-mind contents may support a more comprehensive interpretation of the neural variability. Finally, the cross-cultural invariance should be investigated comparing Dutch and Italian results.

## Conflict of Interest Statement

The authors declare that the research was conducted in the absence of any commercial or financial relationships that could be construed as a potential conflict of interest.
